# Compensatory eye movements during voluntary head and neck movements

**DOI:** 10.3389/fneur.2026.1922717

**Published:** 2026-08-21

**Authors:** Yoshiko Izawa, Hiroko Koda

**Affiliations:** 1 Department of Physiology and Cell Biology, Graduate School of Medicine, Institute of Science Tokyo, Tokyo, Japan; 2 Ueno Sakuragi Otorhinolaryngology Clinic, Tokyo, Japan

**Keywords:** corollary discharge, eye movements, neck movements, vestibuloocular reflex, visual stabilization

## Abstract

We studied compensatory eye movements during passive and active head rotation using the video head impulse test in 5 healthy human subjects. In passive trials, compensatory eye movements occurred with a median latency of 16 ms following the onset of leftward and rightward head movements, and the median velocity gain was 0.94. In contrast, in active trials, the latencies of compensatory eye movements were reduced (median 4 ms) and the velocity gains were increased (median 1.08). We found similar reductions in latency and increases in gain of compensatory eye movements in active trials compared with those in passive trials without a visual target. The present results suggest the potential contribution of corollary discharge of voluntary head and neck movements to compensatory eye movements, consistent with findings in monkey experiments.

## Introduction

Eye movements are coordinated with head and neck movements to acquire a visual target accurately. We have previously reported two types of motor actions of the eyes and neck that were generated by focal stimulation in the frontal eye field (FEF) in trained monkeys (1). In the first type, saccadic eye movements and neck forces were evoked in the same direction, consistent with a gaze shift process. In the second type, slow eye movements and neck forces were evoked in opposite directions, consistent with a visual stabilization process. The slow eye movements and neck forces had near-synchronous latencies and mirror-image characteristics, suggesting that the FEF may be a source of a corollary discharge signal for compensatory eye movements during voluntary neck movements. Corollary discharge provides internal feedback of motor commands (2). Previous studies have supported the idea that compensatory eye movements can be driven by the corollary discharge of neck movements. Bizzi et al. (3) reported anticipatory eye movements to compensate for neck movements in predictive gaze shifts in primates. Compensatory eye movements during head rotation have been shown after loss of vestibular function in monkeys (4, 5) and in humans (6). More recently, compensatory counterrotations of the eyes that are likely to be produced by a corollary discharge of voluntary neck movement have been distinguished from the vestibuloocular reflex (VOR) in guinea pigs (7, 8).

Clinically, voluntary neck movements have been performed in rehabilitation of patients with vestibular dysfunction to improve visual stabilization. The present study was performed to compare compensatory eye movements during passive and active head rotation using the video head impulse test (vHIT) (9) in healthy human subjects. We compared the latencies and velocity gains of compensatory eye movements in passive and active trials, although the vHIT primarily measures velocity gains rather than latencies. This could provide a basis for examining the potential application of a corollary discharge of voluntary neck movement in vestibular rehabilitation.

## Methods

We used the horizontal vHIT (ICS Impulse, Natus, Denmark) to study compensatory slow eye movements during passive and active neck movements in 5 healthy human subjects (3 men, 2 women), aged 29 to 65 years. All experimental protocols were approved by the Medical Research Ethics Committee of Institute of Science Tokyo (I2025-418). A subject was seated facing a wall at a distance of ~1 m in an illuminated room with a structured background. Goggles were placed properly on the subject with a face cushion. The subject was instructed to look straight ahead with and without a visual target. In passive trials, an examiner stood behind the subject, held the subject’s head without touching the goggles or the goggles strap, and turned the subject’s head horizontally by ~10–20° in a brief and unpredictable manner. In active trials, the subject held the head without touching the goggles or the goggles strap and voluntarily generated brief horizontal head turns. This approach was used because voluntary head turns without holding the head tended to have higher velocities than passive head turns. A set of ~20 trials in each direction was analyzed for passive and active head turns, with and without a visual target.

Movements of the right eye were recorded by a camera mounted on goggles at a sampling rate of 250 Hz. Eye position signals were calibrated by measuring the eye movement between two laser beam dots. The velocity of the head movement was measured by a gyroscope mounted on goggles. Eye and head velocities were sampled every 4 ms. Subsequent off-line data analyses were performed with Matlab (MathWorks, Natick, MA). To obtain head acceleration, the head velocity signal was digitally differentiated. Head acceleration was low-pass filtered with a moving average of five data points (−6 dB, 30 Hz). The onset of the head velocity signal was identified as the point in time at which the head acceleration trace crossed the threshold of 3 SDs of the mean of the head acceleration during a 300-ms period ending 300 ms before the peak of head velocity (10). Mean eye velocity and head velocity signals were obtained by aligning the data with respect to the onset of the head velocity signal and calculating the mean within each 4 ms of the data. The onset of eye movements was identified as the point in time at which the mean eye velocity trace crossed the threshold of 3 SDs of the mean of the mean eye velocity during a 300-ms period ending 100 ms before the onset of the mean head velocity (10). The latency of the onset of eye movements was measured from the onset of the mean head velocity. Gain was calculated by the ICS Impulse system as the ratio of the area under the eye movement velocity to the area under the head movement velocity.

We compared the latencies and gains of compensatory eye movements in passive and active trials. Comparisons were conducted using active trials whose peak head velocities fell within ±2 SDs of the mean peak head velocity in passive trials. Although comparisons were initially considered using active trials whose head acceleration signals during the initial 80-ms period of head rotation fell within ±2 SDs of the mean initial head acceleration in passive trials, a pilot analysis in one subject showed comparable results. Accordingly, subsequent analyses were based on trials matched for peak head velocity. Statistical analysis was performed with the Wilcoxon signed-rank test.

## Results

### Trials with a visual target

**Figure 1:**
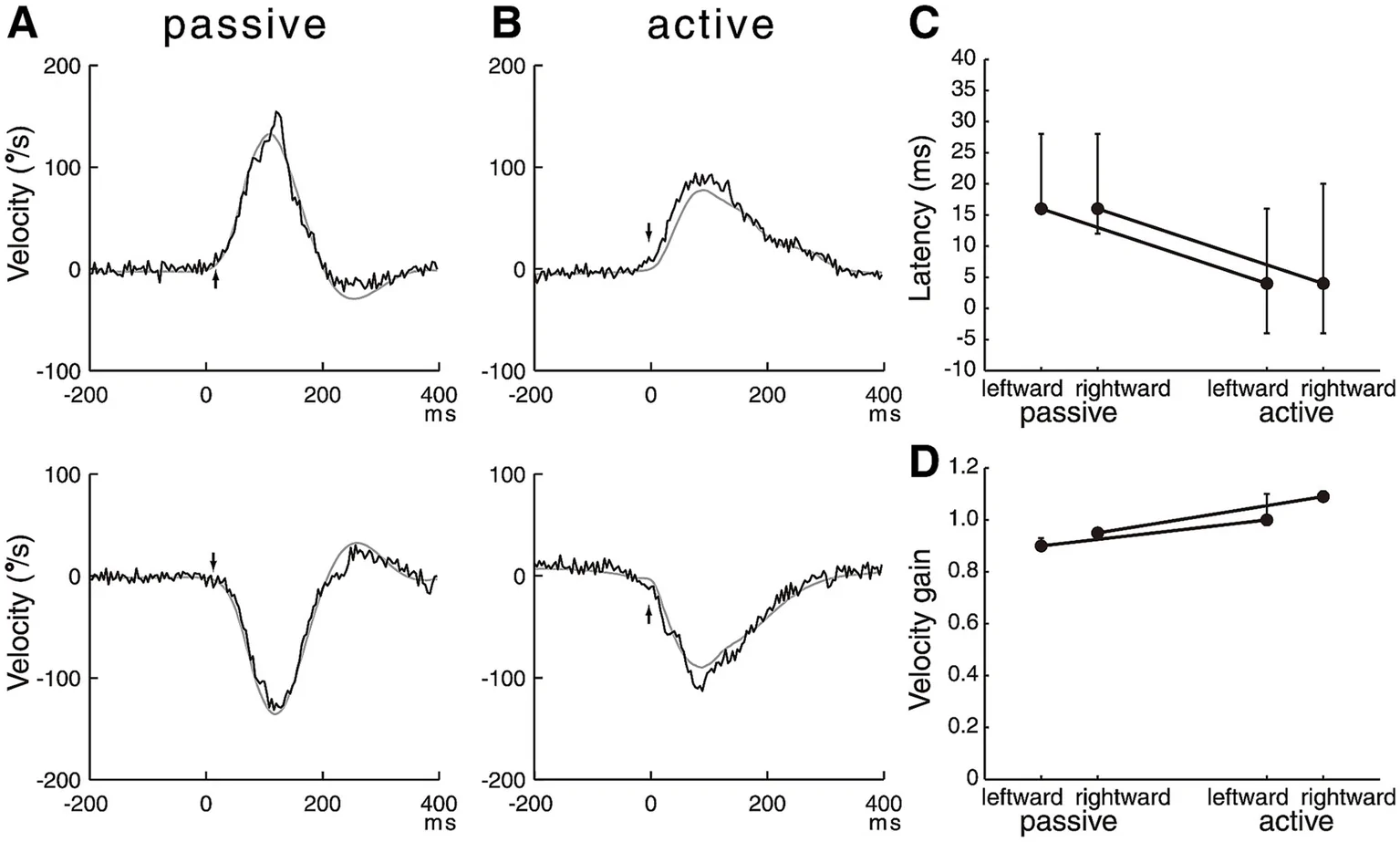
Compensatory eye movements during passive and active head rotation with a visual target. **(A)** Each trace shows the mean horizontal eye (black) and head velocities (gray) during passive leftward (top) and rightward head movements (bottom) in a subject. **(B)** Each trace shows the mean horizontal eye and head velocities during active leftward (top) and rightward head movements (bottom) in the same subject. Traces are aligned with respect to the onset of head movements (abscissa, 0 ms). Arrows indicate the onset of eye movements. Head velocity traces are inverted for comparison. **(C,D)** Summary of the latencies and velocity gains of compensatory eye movements in passive and active trials. Medians and quartiles of the latencies **(C)** and velocity gains **(D)** are plotted for all 5 subjects.

We recorded compensatory eye movements during passive and active head rotation with a visual target placed in front of the subjects. In passive trials, the VOR produced compensatory slow eye movements in the direction opposite that of head and neck movements. In the example shown in Figure 1A, top, compensatory eye movements occurred with a latency of 16 ms following the onset of leftward head movements, and the velocity gain was 0.86. Overall, the latencies of the onset of eye movements with respect to the onset of leftward head movements had a median of 16 ms (interquartile range, 12 ms; Figure 1C). The velocity gains observed during leftward head movements had a median of 0.9 (interquartile range, 0.04; Figure 1D). Similarly, compensatory eye movements occurred with a latency of 12 ms following the onset of rightward head movements, and the velocity gain was 0.90 (Figure 1A, bottom). Overall, the latencies of the onset of eye movements with respect to the onset of rightward head movements had a median of 16 ms (interquartile range, 16 ms; Figure 1C). The velocity gains observed during rightward head movements had a median of 0.95 (interquartile range, 0.03; Figure 1D).

In contrast, in active trials, the latencies of compensatory eye movements were reduced and the velocity gains were increased. In the example shown in Figure 1B, top, compensatory eye movements occurred with a latency of −4 ms from the onset of active leftward head movements, and the velocity gain was 1.13. Overall, the latencies of the onset of eye movements with respect to the onset of active leftward head movements had a median of 4 ms (interquartile range, 20 ms; Figure 1C). The velocity gains observed during active leftward head movements had a median of 1.0 (interquartile range, 0.12; Figure 1D). Similarly, compensatory eye movements occurred with a latency of −4 ms from the onset of active rightward head movements, and the velocity gain was 1.11 (Figure 1B, bottom). Overall, the latencies of the onset of eye movements with respect to the onset of active rightward head movements had a median of 4 ms (interquartile range, 24 ms; Figure 1C). The velocity gains observed during active rightward head movements had a median of 1.09 (interquartile range, 0.04; Figure 1D). The latencies of compensatory eye movements were significantly reduced in active trials compared with those in passive trials (Wilcoxon signed-rank test, data combined from leftward and rightward head movements, *p* < 0.01, *n* = 10). In contrast, the gains of compensatory eye movements were significantly increased in active trials compared with those in passive trials (Wilcoxon signed-rank test, data combined from leftward and rightward head movements, *p* < 0.01, *n* = 10).

### Trials without a visual target

**Figure 2:**
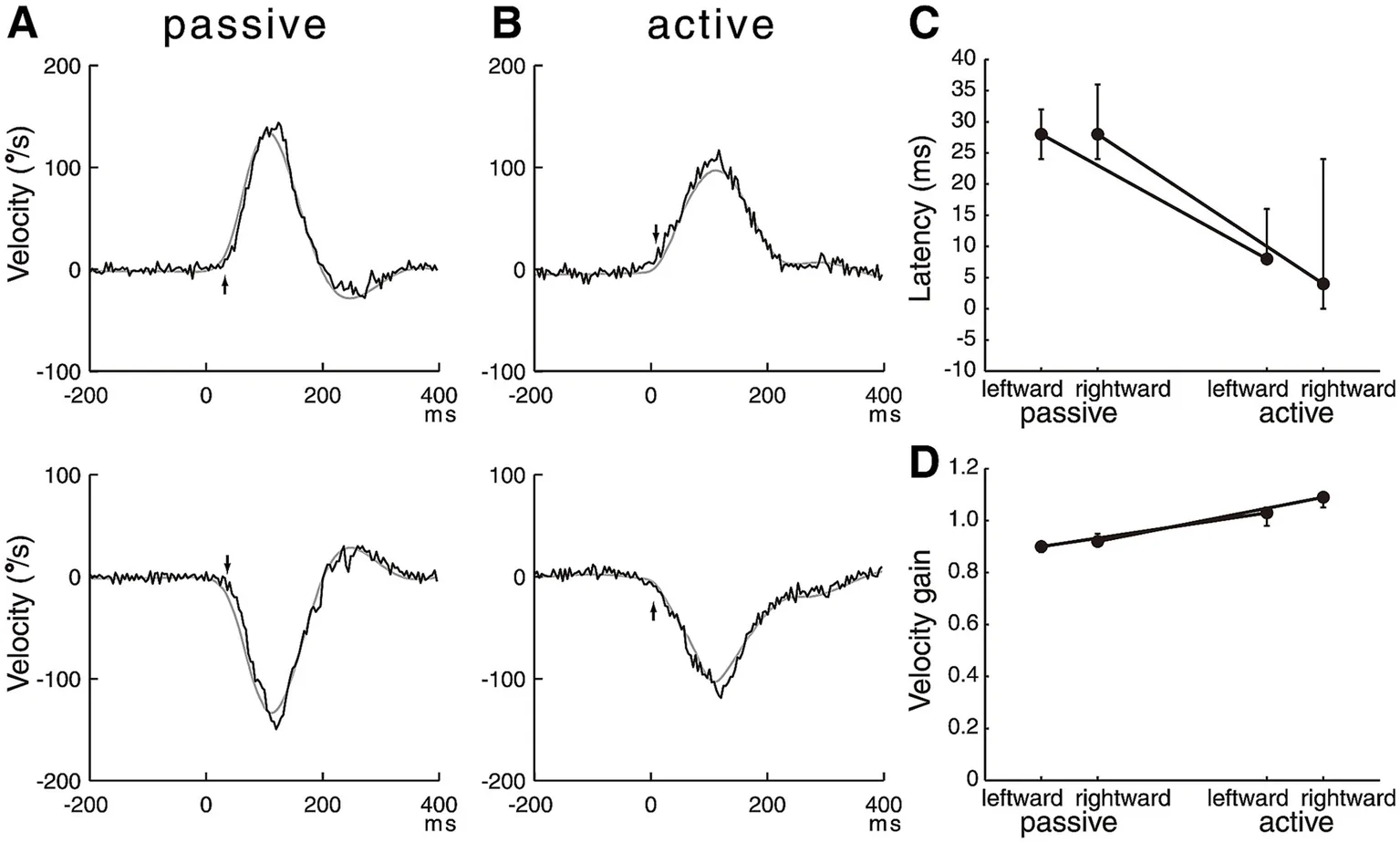
Compensatory eye movements during passive and active head rotation without a visual target. **(A, B)** Traces from the same subject as in Figure 1 **(A, B)**. **(A-D)** Same arrangement as in Figure 1.

We further recorded compensatory eye movements during passive and active head rotation without a visual target. In the example shown in Figure 2A, top, compensatory eye movements occurred with a latency of 32 ms following the onset of passive leftward head movements without a visual target, and the velocity gain was 0.9. Overall, the latencies of the onset of eye movements with respect to the onset of passive leftward head movements without a visual target had a median of 28 ms (interquartile range, 8 ms; Figure 2C). The velocity gains observed during passive leftward head movements without a visual target had a median of 0.9 (interquartile range, 0.03; Figure 2D). Similarly, compensatory eye movements occurred with a latency of 36 ms following the onset of passive rightward head movements without a visual target, and the velocity gain was 0.95 (Figure 2A, bottom). Overall, the latencies of the onset of eye movements with respect to the onset of passive rightward head movements without a visual target had a median of 28 ms (interquartile range, 12 ms; Figure 2C). The velocity gains observed during passive rightward head movements without a visual target had a median of 0.92 (interquartile range, 0.04; Figure 2D).

In active trials without a visual target, the latencies of compensatory eye movements were reduced and the velocity gains were increased. In the example shown in Figure 2B, top, compensatory eye movements occurred with a latency of 8 ms from the onset of active leftward head movements without a visual target, and the velocity gain was 1.05. Overall, the latencies of the onset of eye movements with respect to the onset of active leftward head movements without a visual target had a median of 8 ms (interquartile range, 8 ms; Figure 2C). The velocity gains observed during active leftward head movements without a visual target had a median of 1.03 (interquartile range, 0.07; Figure 2D). Similarly, compensatory eye movements occurred with a latency of 4 ms from the onset of active rightward head movements without a visual target, and the velocity gain was 1.09 (Figure 2B, bottom). Overall, the latencies of the onset of eye movements with respect to the onset of active rightward head movements without a visual target had a median of 4 ms (interquartile range, 24 ms; Figure 2C). The velocity gains observed during active rightward head movements without a visual target had a median of 1.09 (interquartile range, 0.04; Figure 2D). As in trials with a visual target, the latencies of compensatory eye movements were significantly reduced in active trials compared with those in passive trials without a visual target (Wilcoxon signed-rank test, data combined from leftward and rightward head movements, *p* < 0.01, *n* = 10), whereas the gains of compensatory eye movements were significantly increased in active trials compared with those in passive trials without a visual target (Wilcoxon signed-rank test, data combined from leftward and rightward head movements, *p* < 0.01, *n* = 10).

## Discussion

This study analyzed the characteristics of compensatory eye movements during passive and active head rotation with and without a visual target using the vHIT, to understand the contribution of corollary discharge to visual stabilization. We found that the latencies of compensatory eye movements were reduced and the velocity gains were increased in active trials compared with those in passive trials. These results are consistent with previous studies using magnetic search coils (11, 12), while potential influences of goggle slippage and inertia cannot be completely excluded in the vHIT. In active trials, the peripheral vestibular, visual, and neck proprioceptive inputs are nearly comparable to those in passive trials. Therefore, the reduction in latency and increase in gain of compensatory eye movements in active trials may reflect the contribution of corollary discharge of voluntary head and neck movements.

The comparable results obtained for both leftward and rightward head movements suggest that there was no clear difference between abduction and adduction in compensatory eye movements. In active trials with and without a visual target, similar reductions in latency and increases in gain of compensatory eye movements were observed compared with those in passive trials, suggesting that the contribution of visual inputs to these compensatory eye movements was relatively weak. The latencies of compensatory eye movements tended to be longer in trials without a visual target. This may be attributed to suppressive influences of a structured background because the vHIT is typically not performed in darkness. The effect of experimental conditions on compensatory eye movements remains to be examined. It would be important to further examine voluntary head turns without holding the head at velocities comparable to those of passive head turns. A more systematic examination in larger samples is also needed.

Our previous study analyzed compensatory slow eye movements that may be produced by a corollary discharge of motor actions of the neck in monkeys (1). These slow eye movements were dissociated from the VOR by recording neck forces concurrently with eye movements under a head-restrained condition. Compensatory eye movements during active head movements were originally described as predictive eye-head movements in monkeys by Bizzi et al. (3). King and colleagues confirmed the existence of compensatory eye movements whose latency with respect to the onset of voluntary neck movements was essentially zero in guinea pigs with bilateral peripheral vestibular lesions (7, 8). They further reported a model where the anticipatory eye movements compensating for voluntary neck movements work with the VOR in intact animals (13). The concept of corollary discharge goes back to Helmholtz in the nineteenth century, who called the mechanism to eliminate visual motion caused by eye movements the “intensity of the effort of will” (14). As a neural basis for Helmholtz’s idea, Sperry (2) termed central feedback of motor commands “corollary discharge.” An essentially identical concept was termed “efference copy” by von Holst and Mittelstaedt (15).

Clinically, in patients with bilateral peripheral vestibular loss, corrective slow phase eye movements during active head turns are usually insufficient and vary among individuals and across stages of compensation. Rehabilitation of patients with vestibular dysfunction is primarily designed to utilize visual and proprioceptive inputs as factors influencing the compensatory process after vestibular dysfunction. Therefore, utilizing a corollary discharge of voluntary neck movement, in addition to visual and proprioceptive inputs, would help increase the efficiency of the therapy. Vestibular rehabilitation is typically tailored to individual patients and recovery of vestibular function involves different patterns including recovery of slow phase eye velocity and the generation of saccades during head movements (16). There may be individual variation in the weighting of signals that influence the compensatory process after vestibular dysfunction. Assessing the contribution of corollary discharge in individual patients remains an important step. Understanding corollary discharge of voluntary head and neck movements would provide a new perspective on compensatory eye movements after vestibular dysfunction.

## Data Availability

The raw data supporting the conclusions of this article will be made available by the authors, without undue reservation.
